# Association of Early-Life Adversity With Measures of Accelerated Biological Aging Among Children in China

**DOI:** 10.1001/jamanetworkopen.2020.13588

**Published:** 2020-09-21

**Authors:** Ying Sun, Jiao Fang, Yuhui Wan, Puyu Su, Fangbiao Tao

**Affiliations:** 1 Department of Maternal, Child and Adolescent Health, Anhui Medical University School of Public Health, Hefei, Anhui, China; 2 Key Laboratory of Population Health Across Life Cycle, Anhui Medical University, Ministry of Education of the People’s Republic of China, Hefei, Anhui, China; 3 Anhui Provincial Key Laboratory of Population Health and Aristogenics, Anhui Medical University, Hefei, Anhui, China

## Abstract

**Importance:**

A growing body of literature suggests that exposure to early-life adversity (ELA) is associated with accelerated biological aging, offering 1 mechanism through which ELA may be associated with an increased risk for age-related disease. These investigations, however, have been predominantly cross-sectional and focused on adults and females.

**Objective:**

To evaluate associations of threat-related (ie, physical abuse) and deprivation-related (ie, emotional neglect) ELA exposure with cellular and reproductive strategy metrics of biological aging among boys and girls with specific genetic backgrounds around the period of pubertal onset.

**Design, Setting, and Participants:**

In this cohort study, 997 boys and girls in grade 1 to grade 3 from 3 large elementary schools were recruited from Bengbu, Anhui Province, China, and were followed up from March 21, 2016 (baseline; wave 1), for 4 consecutive years, through March 25, 2019.

**Main Outcomes and Measures:**

The outcome was accelerated biological aging in both cellular and reproductive strategy metrics: telomere attrition and age at thelarche (for girls) and testicular maturation (for boys). Multi-informant assessment of exposure to threat-related and deprivation-related ELA was done at baseline (wave 1) and 1-year follow-up (wave 2). The polygenic risk score (PRS) was computed based on 17 single-nucleotide variations for early pubertal timing.

**Results:**

Of the 997 participants (579 girls [58.1%]; mean [SD] age at baseline, 8.0 [0.8] years), 550 (55.2%) reported exposure to threat-related ELA and 443 (44.4%) reported exposure to deprivation-related ELA. Threat-related ELA was associated with onset of thelarche 2.6 months earlier and deprivation-related ELA with onset of thelarche 3.3 months earlier in exposed girls than in unexposed peers; these associations were observed only among girls with a low PRS. Among boys, a similar pattern was found. Threat-related ELA was associated with testicular volume of 4 mL or more 1.4 months earlier and deprivation-related ELA was associated with testicular volume of 4 mL or more 2.3 months earlier than in unexposed peers but only among those with a low PRS. Boys and girls with greater exposure to threats showed a significantly higher percentage of telomere length change during 1-year follow-up, but only among those with low PRS (boys: β = 1.50; 95% CI, 0.80-2.21; *P* < .001; girls: β = 2.40; 95% CI, 1.78-3.05; *P* < .001) and moderate PRS (boys: β = 1.09; 95% CI, 0.43-1.75; *P* = .001; and girls: β = 1.27; 95% CI, 0.77-1.77; *P* < .001). No associations of deprivation-related ELA with percentage of telomere length change were found.

**Conclusions and Relevance:**

This study suggests that the accelerating association of ELA with biological aging might occur at an earlier age and in a genetic background–dependent and dimension-specific manner.

## Key Points

**Question:**

Are there dimension- and sex-specific longitudinal associations between early-life adversity and accelerated biological aging in children with specific genetic backgrounds?

**Findings:**

This cohort study of 997 youths found that both threat- and deprivation-related early-life adversity were associated with earlier age of pubertal onset in boys and girls among those with a low polygenic susceptibility for early puberty. Greater exposure to threat, but not deprivation, was associated with greater telomere attrition among children with low and moderate polygenic profiles.

**Meaning:**

The findings suggest that the accelerating association of early adversity with biological aging might occur at a younger age and act in a genetic background–dependent and dimension-specific manner.

## Teaser

This cohort study evaluates the association of exposure to threat-related and deprivation-related early-life adversity with metrics of biological aging among boys and girls with specific genetic backgrounds around the period of pubertal onset.

## Introduction

Chronic psychosocial stress experienced in childhood is thought to be associated with long-term health and disease risk. In particular, early-life adversity (ELA)—experiences that represent a deviation from the expected environment and require adaptation, including exposure to child abuse, sexual assault, neglect, and chronic poverty—is associated with elevated risk for numerous mental and physical health problems.^1,2,3^

Previous studies have suggested that a potential mechanism for the association between exposure to ELA and this wide range of physical and mental health problems is accelerated biological aging. Exposure to adversity early in life may alter the pace of development, resulting in faster aging. Accumulating evidence suggests that some forms of ELA are associated with accelerated aging using more global metrics of development, including cellular and reproductive strategy indicators. Specifically, ELA has been associated with faster sexual maturation, including earlier pubertal timing and age of menarche,^4,5,6,7,8,9,10^ shorter telomere length (TL) and accelerated telomere attrition,^11,12,13^ and DNA methylation–based epigenetic aging.^5,14,15,16^ Although these studies have often been conducted independently, Belsky and Shalev^17^ offered an evolutionary-developmental framework to include these independent lines of inquiry into a process of accelerated aging whereby long-term health costs are traded for an increased probability of reproducing before dying. This framework holds the promise of advancing understanding of health and development and moves the field from a disease (wear and tear) model to an adaptive (reproductive trade-off) model.

We examined whether ELA is associated with accelerating biological aging in both cellular and reproductive strategy metrics among a group of children followed up for 4 years, focusing on a hallmark of accumulated cellular aging in studies of ELA in youths: accelerated telomere attrition.^13,18^ Short telomeres and telomere shortening rate are associated with greater disease burden and mortality and are a biomarker of the aging process that allows researchers to investigate factors potentially associated with biological aging decades before morbidity and mortality.^19,20,21^

Increasing evidence suggests a secular trend for earlier pubertal timing in both boys^22^ and girls,^23^ which has frequently been associated with various adverse health outcomes over the life span.^24,25,26^ Thus, for our reproductive strategy metric of biological aging, we focused on median age at thelarche and testicular development across 4 repeated annual assessments at baseline (wave 1, in 2016) to wave 4 (in 2019).

We extended prior work by examining the relative associations of deprivation and threat as 2 forms of ELAs with accelerated development. A recent conceptual model posits that 2 core underlying dimensions—threat and deprivation—encompass a wide variety of adverse experiences common in childhood.^27^ Threat includes experiences involving harm or threat of harm, and deprivation involves an absence of expected inputs from the environment, such as cognitive and social stimulation. These dimensions cut across numerous adverse experiences that share the underlying experience of threat or deprivation to varying degrees. We hypothesized that ELA characterized by threat (ie, physical abuse), in particular, would be associated with accelerated biological aging.

Children vary substantially in their susceptibility to many environmental stressors.^28,29,30^ Previous work revealed that chronic stress was associated with earlier age at pubertal onset in a sex-specific and genetic background–dependent manner.^28^ Thus, the present study raised the possibility that anticipated associations of ELA exposure with both telomere erosion and pubertal timing may vary as a function of individual characteristics (ie, genetics).

## Methods

### Study Design

This cohort study used data from an ongoing longitudinal study examining psychosocial factors associated with growth and development in Anhui Province, China. In March 21, 2016 (wave 1), a total of 1263 participants in grade 1 to grade 3 were recruited from 3 large elementary schools in Bengbu, Anhui Province, China, and were followed up annually through March 25, 2019, for physical and pubertal development (wave 2 in 2017, wave 3 in 2018, and wave 4 in 2019). The final sample who had genotype and pubertal development data consisted of 997 children (418 boys and 579 girls) aged 7 to 9 years at baseline. Procedures for the present study were approved by the institutional review boards at Anhui Medical University. We obtained written informed consent from parents and school teachers, as well as written assent from the children. This study followed the Strengthening the Reporting of Observational Studies in Epidemiology (STROBE) reporting guideline.

### Measures

#### Exposure: ELA

A multi-informant approach for assessing ELA exposure was used in our study. Parents were asked questions about the child’s exposure to ELA at baseline (wave 1) and wave 2, including child physical, emotional, or sexual abuse; physical or emotional neglect; parent substance use; parent mental health problems or suicide attempt; marital violence; parental separation; and harsh parenting (eMethods in the Supplement). At wave 2, all children were asked about exposure to ELAs.

Threat-related adversities included 5 specific adversities: physical, emotional, and sexual abuse; experiencing violence in the school; and witnessing domestic violence. Deprivation-related adversities included 5 specific adversities: physical and emotional neglect, low parental educational level (less than a high school degree), low household income (annual household income lower than average in the region), and lack of parental warmth.

Each experience was coded as a binary factor associated with whether the child experienced the adversity or not, and a composite score for each dimension of adversity (threat and deprivation) was generated by summing across all child or parent reports for each type of adversity. Child reports at wave 2 and parent reports at baseline and wave 2 were combined using an “or” rule: each ELA was coded as present if reported by the child or parent. Threat and deprivation composite scores were also categorized as high according to the 90th percentiles, which was 3 for the threat score and 2 for the deprivation score.

#### Pubertal Development

Breast Tanner stage of each girl was assessed by a female pediatric endocrinologist from wave 1 to wave 4. Tanner staging was performed by palpation of breast tissue in addition to visual inspection, following the same protocol of the China Puberty Collaboration Study.^31^ Onset of breast development was defined as attaining breast Tanner stage 2 or greater.

Testicular volume was estimated by a trained and qualified male pediatric endocrinologist from wave 1 to wave 4 through palpation to the nearest 1 mL using a Prader orchidometer.^32^ Onset of testicular development was defined as attaining testicular volume of 4 mL or greater.

#### Telomere Length and Attrition

Telomere length was measured in saliva samples during waves 2 and 3. Genomic DNA was purified from a 500-μL saliva sample collected in the Oragene DNA Kit (DNA Genotek) with the DNA Agencourt DNAdvance Kit (Beckman Coulter Genomics) according to the manufacturer’s instruction. The DNA was quantified by the Quant-iT PicoGreen dsDNA Assay Kit (Life Techonologies) and run on 0.8% agarose gels to check the integrity. The DNA samples were stored at −80 °C.

Telomere length variables were examined for normality and outliers. One TL value was beyond 3 SDs from the mean and was winsorized to the next closest value; no additional transformations were conducted. We chose percentage change (vs a raw change score) as the measure of change independent of baseline length because it adjusts for baseline TL, as recommended by Epel et al^33^; thus, percentage of TL change was used in all analyses and is synonymous with telomere attrition. Percentage of TL change was calculated as a percentage change [(TL2 − TL1) × 100]/TL1 and was considered a continuous variable.

#### Polygenic Risk Score for Early Puberty

The DNA were extracted from buccal swab samples during wave 2 by polymerase chain reaction–restriction fragment length variation and real-time polymerase chain reaction and genotyped using the MassARRAY system (Agena Bioscience) in 1 batch. We chose 17 single-nucleotide variations for decreasing age at menarche and age at voice breaking in men^34,35^ (eTable and eMethods in the Supplement). Polygenic risk scores (PRS) were derived by summing the products of numbers of associated alleles (range, 0-17) and were categorized into 3 groups by tertile: high, moderate, and low.

### Covariates

Models were adjusted for child’s age, body mass index (BMI), early-life factors (delivery mode, birth weight, and gestational age), and lifestyle behaviors (physical activity, consumption of sugar-sweetened beverages, and sleep duration), as well as parents’ current age, height, and weight (eMethods in the Supplement).

### Statistical Analysis

The study sample was characterized using descriptive statistics and frequency distributions in waves 1 through 3. We used linear regression to estimate associations between ELAs (independent variables; threat related and deprivation related) as continuous variables and breast Tanner stage, testicular volume, percentage of TL change, and TL at 1-year follow-up as dependent variables across PRS tertiles for boys and girls, covarying age, pubertal status, BMI, maternal age, maternal BMI, lifestyle behaviors, and early-life factors for the child. Given high co-occurrence of threat and deprivation (*r* = 0.278; *P* < .001), we estimated models that included both dimensions of ELA to evaluate unique associations of each adversity dimension with pubertal development and telomere attrition.

The association of high threat-related and high deprivation-related ELA as categorical variables with the age at thelarche and testicular volume of 4 mL or more over 4 years across PRS tertile was assessed, adjusting for age, BMI, PRS for early puberty, gestational age, birth weight, maternal age and BMI, and other dimensions of ELA. Weibull accelerated failure time models were used to obtain time ratios (TRs) with 95% CIs. All *P* values were from 2-sided tests, and results were deemed statistically significant at *P* < .05. All analyses were conducted in STATA/SE, version 13.1 (StataCorp LLC).

## Results

### General Characteristics

Table 1 presents characteristic of the cohort at baseline and follow-up examinations. Of the 997 participants (579 girls [58.1%]; mean [SD] age at baseline, 8.0 [0.8] years), 218 children (21.9%) had obesity in wave 4. More than half the children (550 [55.2%]) experienced at least 1 form of threat-related ELA exposures, with physical abuse (367 [36.8%]) and emotional abuse (335 [33.6%]) accounting for the greatest portion. The 90th percentile of threat composite score (3 for boys and 2 for girls in our study) was used as the cutoff value for defining high-threat exposure; 128 of 579 girls (22.1%) and 94 of 418 boys (22.5%) reported high-threat exposure.

**Table 1.** Characteristics of the Study Cohort of 997 Children at Baseline (Wave 1) and at Follow-up Examinations (Waves 2-4)

Characteristic	No. of participants	Value
**Sociodemographic characteristic**		
Age, mean (SD), y		
At wave 1	997	8.0 (0.8)
At wave 4	997	11.0 (0.8)
Female, %	579	58.1
BMI, mean (SD)		
At wave 1	997	18.0 (3.0)
At wave 4	997	19.8 (3.9)
Obesity at wave 4, %	218	21.9
Breast Tanner stage at wave 4, mean (SD)	579	3.4 (1.2)
Testicular volume at wave 4, mean (SD), mL	418	5.0 (3.1)
Relative telomere length, mean (SD)		
At baseline (wave 2)	986	1.5 (0.4)
At 1-y follow-up (wave 3)	771	1.4 (0.4)
Change of telomere length, median (IQR), %	771	4.1 (1.0-8.6)
**Adversity exposure**		
Threat composite score, mean (SD)	997	1.04 (1.14)
Physical abuse, %	367	36.8
Emotional abuse, %	335	33.6
Sexual assault, %	35	3.5
Domestic violence, %	113	11.3
Experienced interpersonal violence at school, %	114	11.4
Threat composite score ≥1, %	550	55.2
High threat (>90th percentile), %	222	22.3
Deprivation composite score, mean (SD)	997	0.67 (0.92)
Physical neglect, %	43	4.3
Emotional neglect, %	109	10.9
Less educated mother, %	188	18.9
Low household income, %	164	16.4
Harsh parenting, %	163	16.3
Deprivation score ≥1, %	443	44.4
High deprivation (>90th percentile), %	148	14.8

Abbreviations: BMI, body mass index (calculated as weight in kilograms divided by height in meters squared); IQR, interquartile range.

A total of 443 children (44.4%) reported at least 1 form of deprivation-related ELA exposures, with mother’s low educational level (188 [18.9%]) and low household income (164 [16.4%]) accounting for the greatest portion. The 90th percentile of deprivation composite score (2 for both boys and girls in our study) was used as the cutoff value for defining high-deprivation exposure; 96 of 579 girls (16.6%) and 52 of 418 boys (12.4%) reported high-deprivation exposure.

Median percentage of TL change was 4.1% (interquartile range, 1.0%-8.6%). The mean (SD) relative TL was 1.5 (0.4) in wave 2 and 1.4 (0.4) at 1-year follow-up (wave 3).

### Longitudinal Associations Between ELA Dimensions and Breast Tanner Stage in Girls and Testicular Volume in Boys

Sex-stratified associations of threat-related and deprivation-related ELA exposure with breast Tanner stage in girls and testicular volume in boys along with TL across PRS tertiles after adjustment for age, pubertal status, BMI, early-life factors, lifestyle behaviors, and maternal age and BMI is presented in Table 2. Both threat-related and deprivation-related ELA were associated with advanced breast Tanner stage in girls but only among those with low PRS. Among girls with low PRS, with threat-related ELA exposure, breast Tanner stage increased by 0.14 (95% CI, 0.06-0.23; *P* = .001), and with deprivation-related ELA exposure, breast Tanner stage increased by 0.09 (95% CI, 0.01-0.16; *P* = .02). Among boys with low PRS, with threat-related ELA exposure, testicular volume increased by 0.32 mL (95% CI, 0.14-0.50; *P* < .001), and with deprivation-related ELA exposure, testicular volume increased by 0.41 mL (95% CI, 0.18-0.63; *P* < .001).

**Table 2.** Factors Associated With PRS in Boys and Girls in the Study Sample

Characteristic	PRS					
	Low		Moderate		High	
	β (95% CI)	*P* value	β (95% CI)	*P* value	β (95% CI)	*P* value
Breast Tanner stage						
Threat composite score	0.14 (0.06 to 0.23)	.001	0.05 (−0.01 to 0.11)	.08	0.05 (−0.02 to 0.12)	.19
Deprivation composite score	0.09 (0.01 to 0.16)	.02	0.05 (−0.01 to 0.10)	.10	0.04 (−0.02 to 0.10)	.23
Testicular volume						
Threat composite score	0.32 (0.14 to 0.50)	<.001	0.04 (−0.12 to 0.20)	.63	−0.05 (−0.29 to 0.18)	.66
Deprivation composite score	0.41 (0.18 to 0.63)	<.001	0.08 (−0.10 to 0.27)	.38	−0.02 (−0.32 to 0.27)	.88
**Pecentage of TL change**^**a**^						
Boys						
Threat composite score	1.50 (0.80 to 2.21)	<.001	1.09 (0.43 to 1.75)	.001	0.75 (−0.43 to 1.15)	.13
Deprivation composite score	−0.33 (−1.18 to 0.53)	.45	0.21 (−0.53 to 0.96)	.58	−0.82 (−1.99 to 0.35)	.17
Girls						
Threat composite score	2.40 (1.78 to 3.05)	<.001	1.27 (0.77 to 1.77)	<.001	0.32 (−0.10 to 0.74)	.14
Deprivation composite score	0.15 (−0.53 to 0.82)	.67	0.15 (−0.38 to 0.69)	.58	−0.05 (−0.48 to 0.39)	.84
**Telomere length at 1-y follow-up**^**a**^**^,^**^**b**^						
Boys						
Threat composite score	−0.02 (−0.03 to −0.01)	<.001	−0.03 (−0.05 to −0.02)	<.001	0.005 (−0.004 to 0.02)	.28
Deprivation composite score	0.002 (−0.01 to 0.01)	.68	−0.01 (−0.02 to 0.003)	.23	−0.003 (−0.01 to 0.01)	.61
Girls						
Threat composite score	−0.04 (−0.05 to −0.03)	<.001	−0.02 (−0.03 to −0.01)	<.001	0.001 (−0.006 to 0.008)	.75
Deprivation composite score	−0.001 (−0.01 to 0.01)	.82	−0.003 (−0.01 to 0.003)	.30	0.001 (−0.006 to 0.007)	.94

Abbreviations: PRS, polygenic risk score.

^a^ Adjusted for age, pubertal status, body mass index, early-life factors (birth weight and gestational age), lifestyle behaviors (physical activity, consumption of sugar-sweetened beverages, and sleep duration), maternal age and body mass index, and deprivation composite score.

^b^ Additionally adjusted for telomere length at baseline.

### Longitudinal Associations Between ELA Dimensions on TL Attrition

Sex-stratified associations of threat-related and deprivation-related ELA exposure with percentage of TL change and TL at follow-up across PRS tertiles after adjustment for age, pubertal status, BMI, early life factors, lifestyle behaviors, depressive symptoms, and maternal age and BMI is presented in Table 2. Threat, but not deprivation, was associated with greater percentage of TL change among boys and girls with low and moderate PRS. With threat exposure, the percentage of TL change increased 1.50% (95% CI, 0.80%-2.21%) in boys and 2.40% (95% CI, 1.78%-3.05%) in girls with low PRS and 1.09% (95% CI, 0.43%-1.75%) in boys and 1.27% (95% CI, 0.77%-1.77%) in girls with moderate PRS. In contrast, among those with highest tertiles of PRS, no associations of threat-related ELA with telomere attrition were found (boys: β = 0.75 [95% CI, −0.43 to 1.15]; *P* = .13; girls: β = 0.32 [95% CI, −0.10 to 0.74; *P* = .14).

A similar pattern was found for TL at 1-year follow-up. After controlling for TL at baseline and other covariates, TL at follow-up decreased by 0.02 units with threat exposure among boys with low PRS and by 0.03 units among boys with moderate PRS (Table 2). For girls with low PRS, TL at follow-up decreased by 0.04 units with threat exposure; for girls with moderate PRS, TL at follow-up decreased by 0.02 units. There were no associations between deprivation-related ELA and telomere attrition and TL at 1-year follow-up across different PRS tertiles.

### Longitudinal Associations of ELA Dimensions With Age at Thelarche and Testicular Maturation by PRS

**Figure 1.**
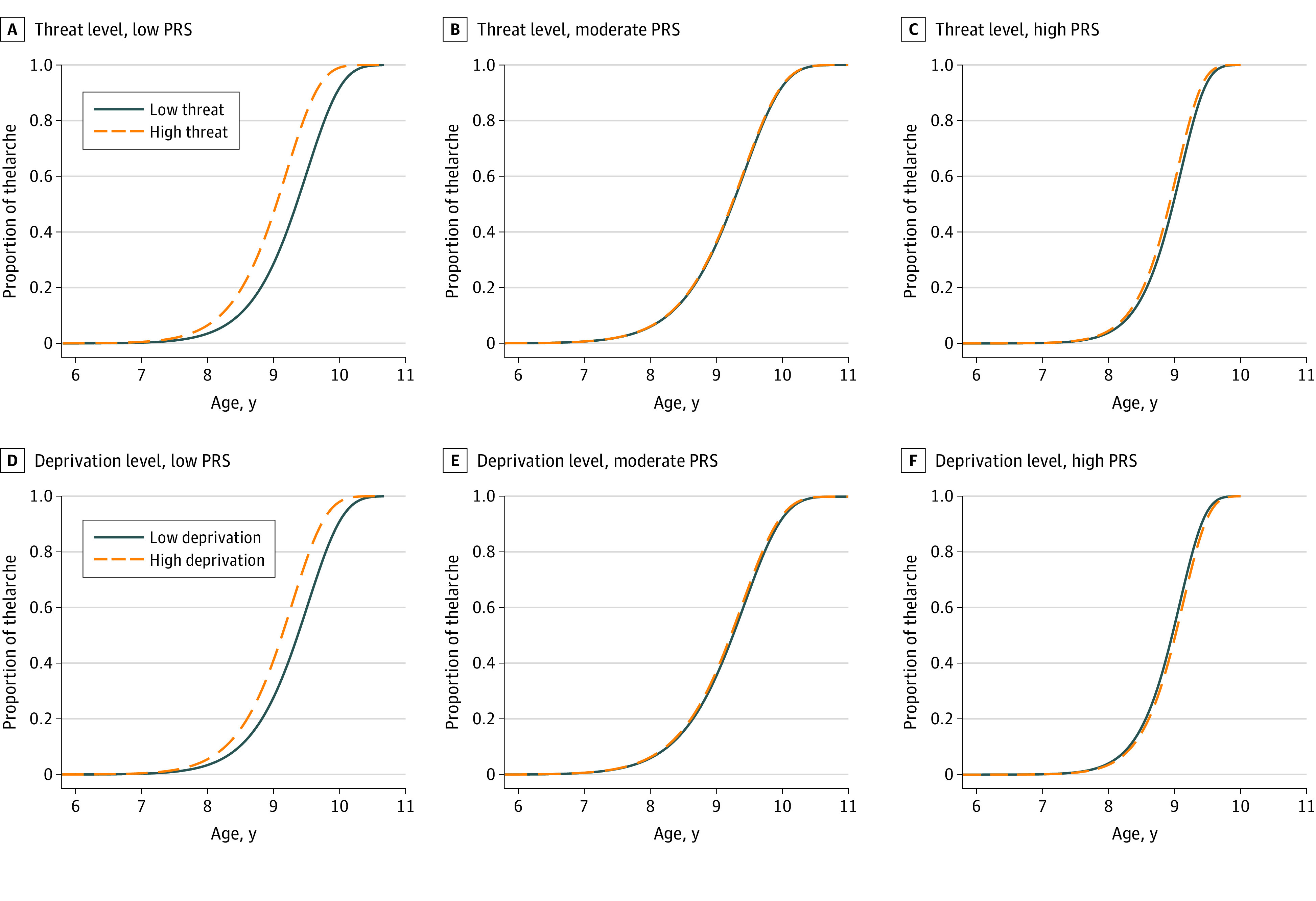
Adjusted Likelihood of Thelarche Between Threat-Related and Deprivation-Related Early-Life Adversity Groups Among Girls With Low, Moderate, and High Polygenetic Risk Scores (PRSs)

After controlling for age, BMI, PRS for early puberty, delivery mode, gestational age, birth weight, maternal BMI, and other dimensions of ELA, high threat was associated with onset of thelarche 2.6 months earlier (9.1 years vs 9.3 years) compared with low threat, and high deprivation was associated with onset of thelarche 3.3 months earlier (9.0 years vs 9.3 years) compared with low deprivation. The associations were observed only among girls with a low PRS (high threat: adjusted TR, 0.96; 95% CI, 0.94-0.99; *P* = .01; high deprivation: adjusted TR, 0.97; 95% CI, 0.96-0.99; *P* < .001) (Table 3 and Figure 1).^36^ No similar association was observed among girls with a moderate PRS (high vs low threat: onset of thelarche, 9.2 years and 9.2 years; *P* = .76; high vs low deprivation: onset of thelarche, 9.1 years and 9.3 years; *P* = .29) and high PRS (high vs low threat: onset of thelarche, 9.0 years and 9.1 years; *P* = .12; and high vs low deprivation: onset of thelarche, 9.1 years and 9.0 years; *P* = .21).

**Table 3.** High Threat-Related and Deprivation-Related Early-Life Adversity and Median Age at Thelarche for Girls and Testicular Volume for Boys by PRS Tertile^a^

Stratum	Thelarche in girls				Testicular volume ≥4 mL in boys			
	No. (%)	Age, median, y	Time ratio (95% CI)	*P* value	No. (%)	Age, median, y	Time ratio (95% CI)	*P* value
Low PRS								
Low threat	109/146 (74.7)	9.3	1 [Reference]	.01	105/137 (76.6)	11.2	1 [Reference]	.005
High threat	37/146 (25.3)	9.1	0.96 (0.94-0.99)		32/137 (23.4)	11.1	0.98 (0.97-0.99)	
Moderate PRS								
Low threat	208/263 (79.1)	9.2	1 [Reference]	.76	131/165 (79.4)	11.2	1 [Reference]	.24
High threat	55/263 (20.9)	9.2	0.99 (0.97-1.02)		34/165 (20.6)	11.1	0.99 (0.98-1.00)	
High PRS								
Low threat	134/170 (78.8)	9.1	1 [Reference]	.12	88/116 (75.9)	10.9	1 [Reference]	.22
High threat	36/170 (21.2)	9.0	0.98 (0.97-1.00)		28/116 (24.1)	10.9	1.01 (0.99-1.01)	
Low PRS								
Low deprivation	123/146 (84.2)	9.3	1 [Reference]	<.001	122/137 (89.1)	11.2	1 [Reference]	.01
High deprivation	23/146 (15.8)	9.0	0.97 (0.96-0.99)		15/137 (10.9)	11.0	0.98 (0.96-0.99)	
Moderate PRS								
Low deprivation	229/263 (87.1)	9.3	1 [Reference]	.29	143/165 (86.7)	11.1	1 [Reference]	.78
High deprivation	34/263 (12.9)	9.1	0.99 (0.98-1.01)		22/165 (13.3)	11.1	0.99 (0.98-1.01)	
High PRS								
Low deprivation	131/170 (77.1)	9.0	1 [Reference]	.21	101/116 (87.1)	10.9	1 [Reference]	.52
High deprivation	39/170 (22.9)	9.1	1.01 (0.99-1.02)		15/116 (12.9)	10.9	0.99 (0.98-1.01)	

Abbreviation: PRS, polygenic risk score.

^a^ Time ratios and 95% CIs were calculated as described by Biro et al^36^ and controlled for age, body mass index, PRS for early puberty, delivery mode, gestational age, birth weight, maternal body mass index, and other dimensions of early-life adversity.

**Figure 2.**
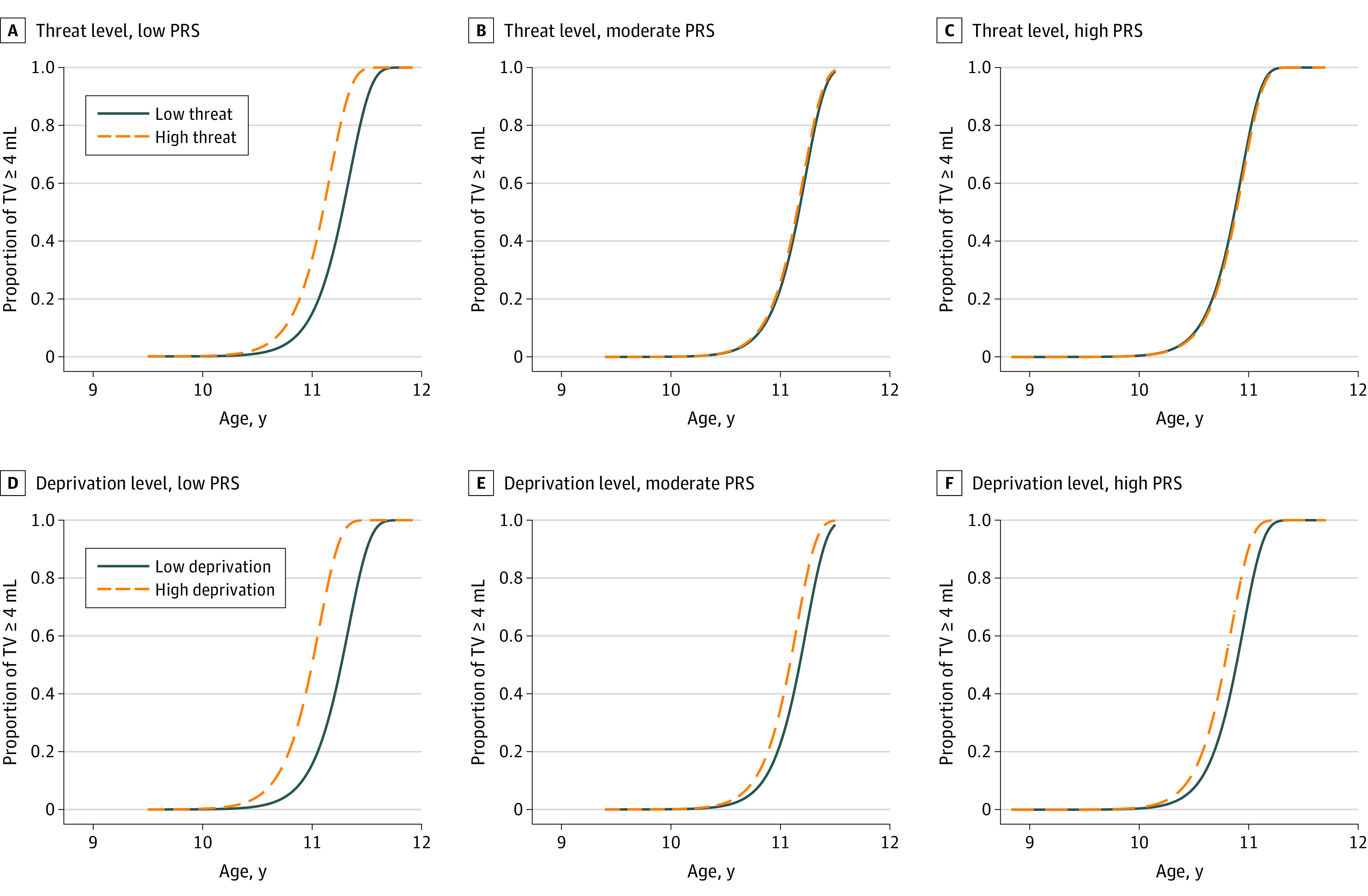
Adjusted Likelihood of Testicular Volume (TV) of 4 mL or More Between Threat-Related and Deprivation-Related Early-Life Adversity Groups Among Boys With Low, Moderate, and High Polygenetic Risk Scores (PRSs)

For boys, after controlling for confounders, compared with low threat-related ELA exposure, high threat was associated with testicular volume of 4 mL or greater 1.4 months earlier (11.1 years vs 11.2 years) and high deprivation was associated with testicular volume of 4 mL or greater 2.3 months earlier (11.0 years vs 11.2 years) among those with a low PRS (high threat: adjusted TR, 0.98; 95% CI, 0.97-0.99; *P* = .005; and high deprivation: adjusted TR, 0.98; 95% CI, 0.96-0.99; *P* = .01) (Table 3 and Figure 2).^36^ For boys with moderate and high PRS, no similar associations were observed.

## Discussion

The study suggests that the association of ELA with accelerated biological aging might act in a genetic background–dependent and dimension-specific manner. Threat-related and deprivation-related ELA exposure were associated with earlier age at thelarche and testicular maturation only among boys and girls with low polygenic susceptibility for early puberty. Our findings also suggest that ELA characterized by threat was associated with a faster rate of telomere attrition over 1-year follow-up among those with low and moderate polygenic susceptibility: each increase in threat events was associated with a 1% to 3% decrease in TL over 1 year. In contrast, ELA characterized by deprivation was not associated with telomere attrition in models adjusting for threat-related ELA.

The findings add to a growing body of literature indicating that ELA exposure may be associated with accelerated development and extend previous findings by further exploring this association in a population of boys and girls with specific genetic backgrounds around the period of pubertal onset (age, 7-9 years) followed up for 4 consecutive years. The sex-stratified analyses revealed that polygenic susceptibility for early puberty could differentially moderate the accelerating association of threat and deprivation with pubertal timing in boys and girls. For example, for children with genetic factors not associated with early puberty, exposure to ELA characterized by deprivation was associated with testicular maturation 2.3 months earlier than in unexposed peers and onset of thelarche 3.3 months earlier than in unexposed peers. This finding is consistent with research on pubertal timing among children with absent fathers,^37^ childhood social disadvantage,^6,38^ and harsh parenting^39^ but contrasts with a previous study by Sumner et al^5^ that indicated that early-life exposure to deprivation was not associated with earlier pubertal development (using Tanner stage measurements). However, that study was cross-sectional and operationalized pubertal timing by using self-reported Tanner staging relative to chronological age only among girls. Given the present findings in a longitudinal sample of boys and girls, it appears that 4-year consecutive assessment of breast and testicular volume development with polygenic susceptibility controlled is important in gaining a more comprehensive understanding of the association between ELA dimensions and early pubertal timing.

Our results showing that a greater amount of threat-related adversities was associated with greater telomere attrition over time replicate findings from a longitudinal study that 2 or more exposures to violence or abuse (ie, maternal domestic violence, frequent bullying exposure, or physical maltreatment) during childhood was associated with greater telomere erosion from 5 to 10 years of age.^13^ We did not find associations of deprivation with telomere attrition, which is consistent with a cross-sectional study from Sumner et al.^5^ In their study of 246 children and adolescents 8 to 16 years of age, early exposure to deprivation, including neglect, food insecurity, and an absence of cognitive stimulation, was not associated with accelerated aging (DNA methylation age). These differential associations should be interpreted with caution, however, because the number of studies examining the type of ELA associated with biological aging was small and the metrics of biological aging were heterogeneous. Greater work is needed to clarify the magnitude and direction of associations for deprivation-related ELA and telomere attrition.

There is evidence to suggest that pubertal timing and cellular aging are highly correlated,^40^ which might reflect a shared mechanism for the association between ELA and accelerated development. The finding dovetails with the claim from Belsky and Shalev^17^ that both telomere attrition and earlier age of pubertal onset can be included in the same evolutionary developmental process whereby long-term health costs are traded for an increased probability of reproducing before dying via a process of accelerated aging. A disease (wear and tear) model emphasizing the pathways leading directly from adversity to dysfunction may miss a fundamental fact about development: the coherent, functional, biobehavioral changes that occur in response to stress over time.^41^ What is routinely characterized as disordered functioning reflects a process of adaptive human development crafted by natural selection because of its correlated reproductive benefits.^17^

The PRS conditioned the associations of ELA with pubertal timing and telomere attrition. These results might provide the explanation of why all children exposed to ELA do not have the puberty-advancing effects highlighted from animal and human studies.^6,7,8,42^ The current finding, at least to some extent, might support the presumption that the developmental processes responsible for accelerated aging under consideration do not likely apply equally to all children.^17^ The findings might also suggest that an individual’s genetic architecture moderates the magnitude and direction of the physiological response to exogenous stressors.^43^

### Limitations

This study has limitations. First, this study represents a relatively small sample, and most of the boys were in the early stages of puberty; the small sample size may have limited the power to detect gene-environment interaction effects. Second, although the genetic sensitivity underlying pubertal timing was found to mediate associations between ELA and pubertal timing and telomere attrition, the genetic loci associated with pubertal timing might have different effect sizes. Third, we recognized that combining across these ELA types by using a composite score is likely an oversimplification of the association of ELA with biological aging. Future research should investigate biological aging after other serious ELA in higher-risk samples. Fourth, TL was not measured in blood but in buccal cells and was only followed up for a relatively short time. A disadvantage of using buccal cells to measure TL is that poor oral hygiene or infection during sampling can alter oral cell composition.^44^ Fifth, future research will benefit from measuring the intensity and duration of multiple adversities across levels, time scales, and domains using repeated assessments of ELA exposures, capturing dynamic cellular aging by tracking multiple hallmarks of aging (eg, mitochondrial function, cellular senescence, and the epigenetic clock).

## Conclusions

This study suggests for the first time, to our knowledge, that the association of ELA with accelerated biological aging might act in a genetic background–dependent and dimension-specific manner. The finding may help refine our understanding of the association of ELA with accelerated biological aging with a focus toward reducing ELA exposures to prevent, slow, and in some cases, reverse the biological aging processes.

## Notes

Supplement. **eMethods.** **eTable.** 17 Single-Nucleotide Polymorphisms (SNPs) for Decreasing Age at Menarche and Age at Voice Breaking in Men in the Present Study
