# Supplementary material for: Association of Early-Life Adversity With Measures of Accelerated Biological Aging Among Children in China
Source: JAMA Netw Open. 2020 Sep 21;3(9):e2013588. doi: 10.1001/jamanetworkopen.2020.13588 (PMC7506517; doi:10.1001/jamanetworkopen.2020.13588)
Supplement: Supplement. — eMethods. eTable. 17 Single-Nucleotide Polymorphisms (SNPs) for Decreasing Age at Menarche and Age at Voice Breaking in Men in the Present Study [file jamanetwopen-e2013588-s001.pdf]

Supplementary Online Content

Sun Y, Fang J, Wan Y, Su P, Tao F. Association of early-life adversity with measures of accelerated biological aging among children in China. *JAMA Netw Open.* 2020;3(9):e2013588. doi:10.1001/jamanetworkopen.2020.13588

eMethods.
eTable. 17 Single-Nucleotide Polymorphisms (SNPs) for Decreasing Age at Menarche and Age at Voice Breaking in Men in the Present Study

This supplementary material has been provided by the authors to give readers additional information about their work.

© 2020 Sun Y et al. *JAMA Network Open.*

eMethods

Exposure: early life adversity (ELA)

Parents filled out a 13-item parental warmth questionnaire adapted by Raudino et al. (2012) [35] from the Child Rearing Practices Report and the Parenting Scale, reflecting the extent to which parents enjoyed spending time together with their children joking and playing expressing affectionate care by hugging, kissing or holding their children (α=0.84). Scores lower the 15th percentile on the scale are considered to be lack of parental warmth.

Parents also reported on total household annual income and highest education level. Total household annual income lower than the average annual salary of an employee working for non-private organizations in urban Anhui Province of China in 2016 (74378 CNY=10656 USD, which was approximately <6198 CNY / 888 USD per month) was indicated as low household income. Parents reported their highest level of education using a 5-point scale (1, did not complete high school; 2, high school; 3, college diploma or trades certificate; 4, undergraduate degree; and 5, graduate degree).

Based on the original Kaiser-CDC ACE Study (Felitti et al., 1998) and the synthesis literatures on ACEs (Finkelhor et al., 2013; Palusci., 2013; Bernstein et al., 2003), exposure to ELA in the present study included physical, emotional and sexual abuse; physical or emotional neglect; parent substance use; parent mental disorder history; marital violence; parental separation. ELA exposure was collected through parents or primary caregivers at Wave 1 and Wave 2, as well as children at Wave 2.

In addition, information regarding violence victimization at school was collected from children at Wave 2, which included any form of physical aggression with intention to hurt (corporal punishment and physical bullying) by adults and other children.

Polygenic Risk Score (PRS) for early puberty

© 2020 Sun Y et al. *JAMA Network Open.*

eTable. 17 Single-Nucleotide Polymorphisms (SNPs) for Decreasing Age at Menarche and Age at Voice Breaking in Men in the Present Study (Elk et al., 2010; Day et al., 2017)

N.	SNP	Chr	Gene	Alleles		Effect size
				Effect	Other	
1.	12617311	2	PLCL1	A	G	0.06
2.	2090409	9	TMEM38B	C	A	0.09
3.	416390	9	RXRA	C	G	0.03
4.	7110373	11	BSX	C	T	0.05
5.	757647	5	KDM3B	A	G	0.05
6.	7642134	3	VGLL3	A	G	0.05
7.	1398217	18	FUSSEL18	G	C	0.05
8.	9408817 †	9	TMEM38B	T	C	0.1
9.	246185	16	MKL2	C	T	0.05
10.	314276	6	LIN28B	C	A	0.14
11.	466639	1	RXRG	T	C	0.08
12.	4840086	6	PRDM13, MCHR2	G	A	0.04
13.	633715	1	SEC16B	C	T	0.05
14.	6438424	3	3q13.32	A	C	0.05
15.	6589964	11	BSX	A	C	0.05
16.	9350100	6	RNF144B-ID4	C	T	0.02
17.	9391253	6	LIN28B	T	A	0.12

© 2020 Sun Y et al. *JAMA Network Open.*

Covariates

Early life indicators

A number of known risk factors for pubertal timing and telomere attrition were collected from the child's primary caregiver during the first wave: gestational age at birth (very early [<33 weeks], somewhat early [33-36 weeks], on time [37-41 weeks], and late birth [42 + weeks]), birth weight, and delivery mode (caesarean section, vaginal delivery).

Child lifestyle factors

Physical activity Physical activity levels were ascertained from the Youth Risk Behavior Survey 2013 (YRBS; Kann et al., 2014) at each wave. Parents (Wave 1) and adolescents (Wave 2-Wave 4) were asked “During the past 7 days, on how many days were your child (you) physically active for a total of at least 60 minutes per day?”.

Sugar-sweetened beverage consumption (SSBs) Sugar-sweetened beverage consumption was assessed though the question ‘During the past 7 days, how many times did your child (you) drink at least 1 serving regular sugar-sweetened sodas, fruit drink, sweetened iced tea, sports /energy drink that contains sugar?’.

Sleep duration Sleep duration was estimated by subtracting self-reported waketime from bedtime, assessed by asking, “On a usual weekday this past week, when did your child (you) go to bed at night?” and “On a usual weekday this past week, when did your child (you) wake up the next morning?”

Body mass index

At each wave, height and weight was measured and body mass index (BMI) was calculated as weight (in kg) divided by height squared (in m). “The China Body Mass Index Curve for Boys and Girls Aged 0-18 Years” is used to convert BMI values into age specific BMI percentile levels (Li et al., 2010).

© 2020 Sun Y et al. *JAMA Network Open.*

eReferences

1. Felitti VJ, Anda RF, Nordenberg D, Williamson DF, Spitz AM, Edwards V, Koss MP, Marks JS. Relationship of childhood abuse and household dysfunction to many of the leading causes of death in adults. The Adverse Childhood Experiences (ACE) Study. Am J Prev Med. 1998; 14(4):245-58.
2. Finkelhor D, Shattuck A, Turner H, Hamby S. Improving the adverse childhood experiences study scale. JAMA Pediatr. 2013; 167(1):70-5.
3. Palusci VJ. Adverse childhood experiences and lifelong health. JAMA Pediatr. 2013; 167(1):95-6.
4. Bernstein DP, Stein JA, Newcomb MD, Walker E, Pogge D, Ahluvalia T, Stokes J, Handelsman L, Medrano M, Desmond D, Zule W. Development and validation of a brief screening version of the Childhood Trauma Questionnaire. Child Abuse Negl. 2003; 27(2):169-90.
5. Elks CE, Perry JR, Sulem P, Chasman DI, Franceschini N, He C, Lunetta KL, Visser JA, Byrne EM, Cousminer DL, et al. Thirty new loci for age at menarche identified by a meta-analysis of genome-wide association studies. Nat Genet. 2010; 42(12) :1077-1085.
6. Day FR, Thompson DJ, Helgason H, Chasman DI, Finucane H, Sulem P, Ruth KS, Whalen S, Sarkar AK, Albrecht E, et al. Genomic analyses identify hundreds of variants associated with age at menarche and support a role for puberty timing in cancer risk. Nat Genet. 2017; 49: 834-841.
7. Kann L, Kinchen S, Shanklin SL, Flint KH, Kawkins J, Harris WA, Lowry R, Olsen EO, McManus T, Chyen D, Whittle L, Taylor E, Demissie Z, Brener N, Thornton J, Moore J, Zaza S; Centers for Disease Control and Prevention (CDC). Youth risk behavior surveillance--United States, 2013. MMWR Suppl. 2014; 63(4):1-168.
8. Li H, Zong XN, Ji CY, Mi J. [Body mass index cut-offs for overweight and obesity in Chinese children and adolescents aged 2 - 18 years]. Zhonghua Liu Xing Bing Xue Za Zhi 2010 31 616-620.

© 2020 Sun Y et al. *JAMA Network Open.*
